# Mesenchymal Stem Cells: An Excellent Candidate for the Treatment of Diabetes Mellitus

**DOI:** 10.1155/2021/9938658

**Published:** 2021-05-28

**Authors:** Qiulan Huang, Yanting Huang, Jianping Liu

**Affiliations:** Department of Endocrinology, The Second Affiliated Hospital of Nanchang University, Nanchang, Jiangxi, China

## Abstract

Mesenchymal stem cells (MSCs) are adult stem cells (ASCs) known for repairing damaged cells, exerting anti-inflammatory responses and producing immunoregulatory effects that can be significantly induced into insulin-producing cells (IPCs), providing an inexhaustible supply of functional *β* cells for cell replacement therapy and disease modeling for diabetes. MSC therapy may be the most promising strategy for diabetes mellitus because of these significant merits. In this paper, we focused on MSC therapy for diabetes.

## 1. Introduction

Diabetes mellitus (DM), a group of metabolic diseases characterized by dysregulated glucose metabolism as a result of insufficient production or effectiveness of the pancreatic hormone insulin, ultimately leads to a series of serious complications and has become a global epidemic with dramatically increasing incidences. Commonly DM falls into two broad pathogenetic categories: type 1 diabetes mellitus (T1DM) and type 2 diabetes mellitus (T2DM). T1DM is characterized by autoimmune destruction of pancreatic *β*-cells resulting in severe insulin deficiency [1], and T2DM results from a combination of insulin resistance and dysfunction of insulin-producing pancreatic *β*-cells [2]. Although conventional available treatments, including oral antidiabetes dugs and exogenous insulin injection, can improve hyperglycemia-related symptoms or temporarily improve insulin sensitivity in target tissues, these treatments reverse neither disease progression nor cellular dysfunction. As a result, exploring effective ways to permanently cure this disease is a priority.

It may be a promising approach to find seed cells to replace damaged or lost *β*-cells to achieve the goal of curing diabetes. According to previous reports, pancreatic or islet cells have been transplanted into patients to replace islet cells with loss of function and then successfully improve the insulin requirement [3, 4]. However, its application is limited by the lack of donors, immune rejection, and severe postoperative complications [3, 5, 6]. Fortunately, MSCs, known for their lower immunogenicity and self-renewal ability, can be induced into insulin-producing cells (IPCs) and have attracted significant attention for the treatment of DM. Importantly, MSCs are also well known for their immunomodulatory and anti-inflammatory capabilities, which have been widely used to treat immune diseases such as severe aplastic anemia [7, 8], multiple sclerosis [9, 10], and nonalcoholic steatohepatitis [11–13]. Moreover, MSCs can secrete some cytokines, which is beneficial for improving the microenvironment of the pancreas, thereby protecting islet function and even reversing damaged cell function.

## 2. IPCs Transplantation

Stem cells can spontaneously differentiate into IPCs in vivo or in vitro, which is one of the key mechanisms by which MSCs treat DM. However, spontaneous differentiation efficiency is extremely low [14]. Numerous studies have chemically [15–17] or genetically [18–20] induced MSCs into IPCs by effectively applying small molecules or genetic engineering to improve differentiation efficiency. Genetic engineering schemes are highly efficient, expensive, cumbersome, and time-consuming, and most of them use viruses as vectors, which are teratogenic and form tumors. Chemical induction is an indirect differentiation application using small-molecule compounds such as activin A, nicotinamide, trichostatin A, and *β*-mercaptoethanol, which are nonimmunogenic and easier to synthesize, standardize, and preserve [21]. The published literature on the differentiation of MSCs into IPCs which has been proved to respond well to glucose stimulation in vitro and in vivo is shown in Table 1. These induction protocols usually mimic the pancreatic developmental microenvironment with small-molecule compounds and gradually induce MSCs to differentiate into IPCs in stages. Researchers have variously optimized the schemes to improve induction efficiency. For example, Karimi et al. [22] demonstrated that vildagliptin (VG) combined with activin A, nicotinamide, fibroblast growth factor, epidermal growth factor, N2, B27, etc. elevates the differentiation of adipose-derived MSCs (AD-MSCs) into IPCs. Insulin release from VG-treated AD-MSCs showed a nearly 3.6-fold increase when exposed to high-glucose medium, and the percentage of insulin-positive cells in the VG-treated cells was approximately 2.9-fold higher than that in the untreated AD-MSCs. Mahmoud et al. [23] used activin A, nicotinamide, and other compounds to directly induce BMSCs into IPCs, which can express transcription factors and pancreatic hormone genes similar to those expressed by pancreatic islets, and further transplantation into nude diabetic mice could maintain euglycemia in diabetic mice for 3 months. Later, their team transferred the IPCs packaged in a capsule device into diabetic dogs, which also achieved remarkable results [24]. Many successful cases have effectively controlled blood glucose in animal models of diabetes by IPC transplantation [15, 25], which offers a promising treatment choice for DM. In addition, some researchers [23, 26] have proven that transplantation of IPCs is more effective than MSCs in controlling blood sugar.

Unfortunately, there are many issues that need to be addressed. First, the cell survival time is shortened due to chemical toxicity after MSCs are induced into IPCs. Moreover, some researchers have considered that there is no evidence for significant transdifferentiation of bone marrow into pancreatic cells in vivo [14]. In addition, in Hassanin et al.'s [27] opinion, despite the weak immunogenicity of IPCs derived from MSCs in vitro, they could still induce an immune response or different degrees of inflammatory response.

## 3. Clinical MSCs Transplantation

### 3.1. Clinical Study of MSCs Therapy for T1DM

T1DM is a multifactorial disorder characterized by T cell-mediated autoimmune destruction of *β*-cells [34]. In particular, T1DM is a silent killer of *β*-cells that only occurs when the *β*-cell mass is reduced to less than 20%, resulting in the inability to secrete insulin [35]. As a result, daily insulin injections are needed for T1DM as a life-saving measure. Insulin is required for regulating the rate at which cells are able to uptake and metabolize glucose and is thus critical for determining how cells store and utilize fuels [36]. Unfortunately, it quickly became clear that the delivery of exogenous insulin via subcutaneous injections was nonphysiological and crude. Moreover, exogenous insulin cannot respond to changeable blood glucose levels in vivo, although insulin saves the lives of T1DM patients. In terms of physiology, pancreatic *β*-cells can release insulin physiologically in response to changes in blood sugar in glucose-stimulated insulin secretion (GSIS) and cannot be replaced by exogenous insulin injections to balance the glucose level, which is exquisitely adjusted by the islet cells. The imbalance demonstrated itself in the development of hyperglycemia-driven micro- and macrovascular complications over time. Moreover, hypoglycemia, a dangerous acute complication, is still a frequent occurrence due to delayed insulin action [37, 38].

To improve this dilemma, many scientists are working on MSC therapy for diabetes. Some clinical projects have been approved. As of March 2021, there are 27 clinical studies on MSCs in the treatment of T1DM in the clinical trials registry (http://www.clinicaltrials.gov), most of which were umbilical cord MSCs (UC-MSCs), namely, Wharton's jelly MSCs (WJ-MSCs) and bone marrow-MSCs (BM-MSCs) for the treatment of diabetes, and another was menstrual blood-derived MSCs and one detached tooth-derived MSCs for T1DM. This review does not discuss the treatment of DM complications as its main subject. The published literature except for the treatment of DM complications for evaluating transplanted MSCs for T1DM is shown in Table 2. Carlsson's study [39] was an open single-center randomized pilot study to first evaluate the safety and efficacy of autologous BM-MSCs in the treatment of recently diagnosed T1DM. All patients who received autologous BM-MSCs treatment during the follow-up period tolerated treatment well, with no adverse events. In addition, most patients randomized to the MSC treatment group increased their capacity for C-peptide response to the mixed-meal tolerance test (MMTT) during the study period, with increased delta values for both peak C-peptide response and MMTT C-peptide response to the MMTT when compared with the control group [39]. However, in this study, there were no significant improvements in C-peptide peak values and C-peptide, HbA1c, and insulin requirements before treatment with MSCs and only a significant improvement compared with the control group. Hu et al. [40] and Cai et al. [41] reported that UC-MSCs significantly improved the patient's metabolic index after treatment, which is encouraging and promising. None of these studies showed significant side effects, demonstrating the safety of MSC therapy.

However, these studies have a small sample size and lack a multicenter study, which cannot be applied as a specification in clinical practice. Although these studies fully demonstrate the short-term safety, effectiveness, and inspiration of MSC therapy for T1DM, they have small sample sizes and lack multicenter research to be used on a large scale. The safety and efficacy of MSCs in the treatment of T1DM have been validated in animals and are currently in the small-sample clinical trial phase. Many studies have been initiated and are in the process of research.

### 3.2. Clinical Study of MSC Therapy for T2DM

T2DM is caused by immune dysfunction and inflammation, which are likely key factors in the development of insulin resistance in T2DM. MSC therapy has been reported to be expected to effectively cure diabetes and ameliorate insulin resistance, which has already been validated in animal trials [49–51]. In addition, there are 21 clinical studies on MSC therapy in the ClinicalTrials registry (http://www.clinicaltrials.gov) as of March 2021, in which 7 studies were completed. The published literature for evaluating transplanted MSCs for T2DM is shown in Table 3. In these published studies, it has been confirmed that MSC therapy can effectively reduce FBG, PBG, and HbA1c, reduce insulin requirements, and improve insulin resistance in follow-up time, proving that MSC therapy has a significant effect in clinical trials. In these clinical evaluations, there are some side effects, including fever, subcutaneous hematoma, nausea, vomiting, headache, and minor hypoglycemia; fortunately, they can be relieved after symptomatic treatment without serious complications or side effects. However, these studies were too short for follow-up evaluations, as they were always only 12 months, to assess long-term side effects and complications. Despite many challenges, the current results, which are reassuring and encouraging, demonstrate that MSC therapy is a promising method for T2DM and suggest a new era of diabetes treatment.

## 4. Mechanisms of MSC Therapy for DM

### 4.1. Differentiation into IPCs

MSCs can be induced to differentiate into IPCs, which is the earliest discovered mechanism for treating diabetes; as a result, MSCs can be used to replace the damaged or hypofunctional *β*-cells to secrete insulin for hypoglycemic treatment, which is the most direct and fundamental treatment for diabetes. Moreover, it is also the basic mechanism by which all stem cells treat diabetes. Many induction protocols have been developed to stably induce MSCs to differentiate into IPCs in vitro, which can effectively control blood sugar within the normal range. After transplanting IPCs into mice, it was found that the content and release profile of human insulin in diabetic mice were similar to those of normal mice, while diabetic mice release very little endogenous insulin [23, 52]. This shows that the IPC cluster derived from human MSC differentiation replaced impaired islet cells to release insulin in mice, fully proving that IPCs derived from human MSCs can treat diabetes. However, there are still many problems to be resolved in the differentiation of MSCs into IPCs. For example, the induced cells may cause immune rejection, some compounds commonly used to induce differentiation can damage the cells, and the risk of tumorigenicity caused by virus-mediated differentiation [53–56]. Although MSCs has immunomodulatory effect to inhibit immune rejection, some studies have shown that MSCs lose immunoprivileged state and acquire immunogenicity after differentiating into IPCs [53], smooth muscle cells, and endothelial cells [54]. Previous study showed that the use of viral vectors to introduce exogenous genetic material into cells carries the risk of tumorigenesis [55]. As for chemically induced differentiation, some small molecular compounds commonly used to induce stem cells to differentiate into IPCs, such as TSA, can increase the apoptosis rate of cells [56]. Therefore, it is important to improve the existing induction methods and find new induction methods to obtain effective and safe IPCs.

### 4.2. Amelioration of Insulin Resistance

Insulin resistance (IR) is an abnormal physiological state in which the body's response to insulin secreted either endogenously or exogenously is decreased. IR is implicated in the pathogenesis of T2DM [57]. To achieve a good effect of lowering blood glucose, it is necessary to improve IR clinically. As research progressed, researchers found that the effect of MSCs on diabetes was not just mediated by the secretion of insulin; most MSCs could ameliorate IR by their anti-inflammatory potential. In 2012, it was first reported that MSC treatment could improve insulin sensitivity in T2DM [58]. MSC treatment resulted in the expression of GLUT4, phosphorylated insulin receptor substrate-1 (IRS-1), and increased protein kinase B (AKT) in insulin target tissues [58]. GLUT4, IRS-1, and AKT are essential for insulin signaling and glucose uptake [59–61]. Decreased expression of GLUT4 and dysregulation of IRS-1 and AKT phosphorylation indicate IR. Similarly, Sun et al. [62] discovered that in the presence of UC-MSCs, knockdown of NLRP3 or IL-1*β* partially improved palmitic acid and lipopolysaccharide-induced insulin signaling impairments. Simultaneously, UC-MSC infusion significantly ameliorated hyperglycemia in T2DM rats and decreased inflammatory activity, which resulted in improved insulin sensitivity in insulin target tissues. Gao et al. [63] improved insulin resistance in T2DM rats by overexpressing apelin in MSCs. During this process, it was found that the secretion of the inflammatory factors IL-6 and TNF-*α* significantly decreased, whereas the secretion of the anti-inflammatory factor adiponectin significantly increased. The inflammatory cytokines IL-6 and TNF-*α* have been implicated in insulin resistance [61]. Moreover, Xie et al. and Gao et al. [64, 65] used UC-MSCs to differentiate macrophages into M2 cells with an anti-inflammatory phenotype to improve IR in T2DM mice or rats. In addition, Zhang et al. [66] proved that M2 cells ameliorate IR by remodeling inflammatory/macrophage homeostasis in obese mice. Further analysis showed that proinflammatory phenotype M1 cells stimulated UC-MSCs to increase the expression of IL-6, a molecule upregulating IL4R expression, promoted phosphorylation of STAT6 in macrophages, and eventually polarized macrophages into the M2 phenotype [64]. Changes in the levels of IL-6 in these two studies seem contradictory. However, this also explains the process of inflammation. When IR is present, MSCs increase the expression of IL-6 to promote the differentiation of M1 to M2 macrophages, thereby ameliorating IR and reducing the levels of inflammatory cytokines. Therefore, IL-6 expression levels increase in the early stage of treatment with MSCs (24 hours [64]), while IR is ameliorated and IL-6 levels decrease in the late stage of transplantation (after 42 days of transplantation [63]). These results prove that MSCs ameliorate insulin resistance by regulating the release of inflammatory factors, upregulating anti-inflammatory factors and/or downregulating inflammation.

### 4.3. Actions on *β*-Cells

Some studies have found that MSCs home to the pancreas after infusion and differentiate into islet *β*-cells [67]. However, very few cells can be located in the pancreas of diabetic animals after infusion, and only a small portion differentiate into islet *β* cells, which is far from sufficient to explain the large number of new *β*-cells induced by cell therapy [68]. Therefore, there may be other mechanisms by which MSCs promote *β*-cell regeneration.

The study found that human bone marrow mesenchymal stem cells (hBM-MSCs) treatment increased the volume, number, and insulin immunoreactivity of diabetic mice. In addition, the study also observed that many pancreatic islets in diabetic mice treated with hBM-MSC germinated from the pancreatic duct, indicating that hBM-MSCs promote the repair and regeneration of endogenous pancreatic islets [69]. Similarly, studies by Hao et al. have shown that a single injection of BM-MSCs infusion can reduce the morphological and structural damage of islets, significantly restore the proportion of insulin-positive cells per islet, and increase the number of islets, though the numbers were still lower than normal. After multiple injections of BM-MSCs, the damaged islets gradually recovered to near normal levels, and the number of islets and the proportion of insulin-positive cells per islet also returned to almost normal levels [70]. In addition, coculture of human pancreatic islets with human adipose-derived MSCs overexpressing betatrophin can induce pancreatic islet proliferation, *β*-cell-specific transcription factor expression, and insulin production under the stimulation of glucose or KCl and Arg [71].

**Figure 1:**
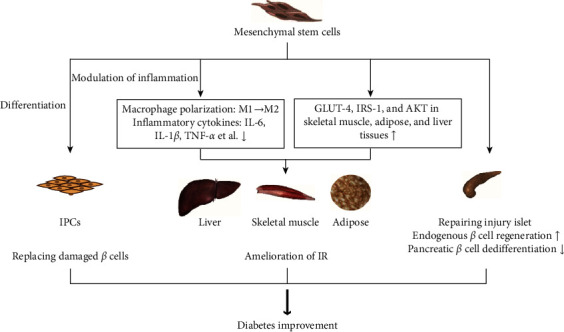
Mechanisms of MSC therapy for DM.

In addition to promoting islet proliferation and repair, MSCs also inhibit cell dedifferentiation. Pancreatic *β*-cell dedifferentiation means that islet *β*-cells lose their specific phenotype, resulting in reduced endocrine function, which is an important mechanism of T2DM [72]. Animal experiments have shown that in a mouse model of T2DM, the specific identity transcription factors Nkx6.1 and Pdx1 of pancreatic *β*-cells decrease, while the progenitor cell markers Neurogenin 3 (Ngn3) and OCT4 increase, affecting the function and number of pancreatic *β*-cells [73]. Clinical trials have also confirmed that pancreatic *β* cells in T2DM patients have undergone significant dedifferentiation [74]. Wang et al. found that MSCs can alleviate *β*-cell dysfunction by reversing *β*-cell dedifferentiation in an IL-1Ra-mediated manner. The results of this study showed that increased expression of proinflammatory cytokines in human T2DM pancreatic islet cells activates MSCs to secrete an IL-1R antagonist (IL-1Ra), which acts on inflamed pancreatic islets and reverses *β*-cell dedifferentiation. In vivo experiments further showed that treatment of db/db mice with MSCs can improve blood sugar in db/db mice and reverse the dedifferentiation of pancreatic *β*-cells [75]. However, there are still few published studies on the dedifferentiation effect of MSCs on diabetic pancreatic *β*-cells. Further in-depth exploration will help researchers understand the mechanism of MSCs in the treatment of diabetes and provide new ideas for MSCs in the treatment of diabetes.  Figure 1 summarizes the various mechanisms of MSC therapy for DM.

## 5. Conclusions

MSCs have an immunosuppressive effect and secrete a variety of cytokines, improve the microenvironment of diabetic patients, target insulin-resistant tissue, ameliorate the metabolic disorder of islet damage, and protect and regenerate islet *β*-cells, thereby reducing blood sugar levels. Due to the main immune mechanism of T1DM, MSCs can also effectively cure type 1 diabetes, precisely because of the immune regulation of MSCs. Among the clinical studies of cell therapy for type 1 diabetes, nearly half of them are studying MSC therapy (http://www.clinicaltrials.gov), which is sufficient to show that MSCs are likely to be an excellent candidate for the treatment of T1DM in the future. Moreover, for T2DM, MSCs can effectively ameliorate IR and anti-inflammatory effects and can partially restore *β*-cell function, which can allow T2DM patients to control blood glucose without using any oral antidiabetic medications or exogenous insulin for a certain period of time.

Although clinical trials of MSC therapy are effective and have few side effects, there are still many problems that need to be solved before MSCs can be applied in the clinic. First, which is the better approach, transplanted MSCs or IPCs? Both methods work effectively regardless of whether MSCs or IPCs are transplanted into patients. However, Anshu Sharma [76] believes that because MSCs have immunoregulatory capabilities over IPCs, autologous MSCs grown in high-glucose medium for 10 to 13 passages may have beneficial effects in individuals at high risk of developing type 1 diabetes. From the clinical trials above, it can be found that the main clinical applications are UC-MSCs and BM-MSCs. MSCs derived from Wharton's jelly of human umbilical cord are generally from healthy and pregnant women with informed consent, and most bone marrow-derived cells are from patients themselves. In previous research, Katarzyna et al. [77] reported that dysfunction of MSCs isolated from T2DM patients may limit their potential therapeutic use as a result of oxidative stress and autophagy. This means that cells of autologous origin, similar to autologous BM-MSCs, will affect the efficacy of cells due to the influence of their own metabolic disorders. Conversely, MSCs allogeneic transplantation, in some cases, such as differentiation which may eliminate the immunoprivileged state of MSCs, may cause immune rejection, even though MSCs have more or less immunomodulatory capabilities [53, 54, 78, 79]. Therefore, which one is the best? The effects need to be compared with large samples from multiple centers, and then a standard clinical treatment needs to be developed, including specifications, injection site, injection method, injection dose, and other variables.

## Figures and Tables

**Table 1:** Protocols that induce MSCs into IPCs.

Cell source	Induce MSCs into IPCs	Transplantation way and results
*Rat BM-MSCs* [26]	Stage 1, 6 days: 10 ng/ml bFGF, 10 ng/ml EGF, 2% B27	Transplantation way: injected into intraperitoneal
	Stage 2, 6 days: 10 ng/ml HGF, 10 ng/ml b-cellulin, 10 ng/ml AA, 10 mmol/L NA, 2% B27	Results: IPC transplantation improved insulin level better than MSC transplantation

*Human BM-MSCs* [24];	Stage 1, 3 days: 55 nmol/L TSA, serum-free DMEM	Transplantation way: cells were loaded in 2 TheraCyte capsules and transplanted under the rectus sheath

*Mice BM-MSCs* [28]	Stage 2, 7 days:10 nmol/L GLP-1, 10% FBS, DMEM : DMEM/F12	Results: the transplanted cells were glucose-responsive and insulin-secreting. Four weeks after transplantation, blood sugar values became normal

*Rat AD-MSCs* [29]	Stage 1, 2 days: 10 mmol/l NA, 0.5 mmol/l *β*-mercaptoethanol and serum-free high-glucose DMEM (25 mmol/l)	Transplantation way: transplanted into the distal tip of the spleen
	Stage 2, 26 days: 30 ng/ml FGF, 10 mmol/l NA and serum-free high-glucose DMEM (25 mmol/l)	Results: IPC transplantation significantly reduced the glucose level. And IPCs were indeed responsive to a glucose challenge in vivo

*Human UC-MSCs* [30]	Stage 1, 7 days: CMRL1066 medium containing 10% FBS, 1% PSA, 100 ng/ml of *β*-nerve growth factor, 4 nM AA, 10 mM NA, and 25 ng/ml EGF	Transplantation way: injected through a retroorbital vein
	Stage 2, 7–10 days: the culture medium was changed to DMEM/F12, and the other components were the same as those in stage 1	Results: IPC transplantation decreased blood glucose, improved glucose tolerance, increased body weight, and prolonged the survival time of NOD mice. And IPCs containing human C-peptide and human nuclei were located in the liver
	Stage 3, 17 days: 10 mM NA, ITS, and 10 ng/ml bFGF	

*Human BM-MSCs* [23]	Stage 1, 2 days: serum-free, glucose-rich DMEM (25 mmol/L) containing 0.5 mmol/L *β*-mercaptoethanol	Transplantation way: inserted under the renal capsule
	Stage 2, 8 days: serum-free, glucose-rich medium containing 1% nonessential amino acids, 20 ng/ml bFGF, 20 ng/ml EGF, 2% B27 supplement, and 2 mmol/L L-glutamine	Results: IPC treatment resulted in control of nude diabetic mice diabetic status for 3 months
	Stage 3, 8 days: serum free, glucose-rich DMEM containing 10 ng/ml betacellulin, 10 ng/ml AA, 2% B27 supplement, and 10 mmol/L NA	

*Human BM-MSCs* [31]	Stage 1, 3 days: DMEM, 55 nmol/L TSA	Transplantation way: implanted beneath the renal capsule
	Stage 2, 7 days: high-glucose (25 mmol/L) medium containing a 1 : 1 ratio of DMEM : DMEM/F12, 10% FBS, and 10 nmol/L GLP-1	Results: diabetic mice became euglycemic 8 ± 3 days after transplantation. The results of the oral glucose tolerance test were normal

*Rat BM-MSCs* [32]	Stage 1, 2 days: DMEM low-glucose medium containing 10 mmol/L NA,0.5 mmol/L 2-mercaptoethanol, and 5% FBS	Transplantation way: injected via tail veins
	Stage 2, 24 hours: serum-free DMEM high-glucose medium containing 0.5 mol/L 2-mercaptoethanol, 10 mmol/L NA, 5% FBS, and10 ng/Ml AA	Results: IPC therapy significantly improved the body weight and serum insulin, alpha-amylase, adiponectin, creatinine, total cholesterol, triacylglycerol, IL-6, TNF-*α*, liver L-malonaldehyde, and glycogen levels in the STZ-induced diabetes model
	Stage 3, 8 days: DMEM-HG medium containing 20 ng/mL bFGF, 20 ng/mL EGF, 2 mmol/L L-glutamine, 5% FBS, and10 mmol/L NA	

*Human UC-MSCs* [33]	Stage 1, 2 days: DMEM/F12 (1 : 1) with 17.5 mM glucose, 1% fatty acid-free BSA Cohn fraction V, 4 nM AA, 1% PSA, 1× ITS-X (ITS-X; 5 mg/L insulin, 5 mg/L transferrin, and 5 mg/L selenium), and 50 *μ*M 2-mercaptoethanol	Transplantation way: injected via the portal vein
	Stage 2, 2 days: DMEM/F12 (1 : 1) with 17.5 mM glucose, 1% BSA, 1% PSA, ITS-X, and 0.3 mM taurine	Results: IPC treatment increased serum insulin and C-peptide level and improved glucose tolerance
	Stage 3, 6 days: DMEM/F12 (1 : 1) with 17.5 mM glucose, 1.5% BSA, ITS-X, 1% PSA, 3 mM taurine, 100 nM GLP-1, 1 mM NA, and 1× nonessential amino acids	

VG : vildagliptin, bFGF: basic fibroblast growth factor, HGF: hepatocyte growth factor, EGF: epidermal growth factor, AA: activin A, NA: nicotinamide, TSA: trichostatin A, DMEM: Dulbecco's Modified Eagle's Medium, GLP-1: glucagon-like peptide 1, FBS: fetal bovine serum, ITS: insulin transferrin selenium, and PSA: penicillin/streptomycin/amphoteric B.

**Table 2:** Clinical studies of MSC therapy for T1DM.

Publication	Cell resource	Injection method	Injection dose	Follow-up time	Efficacy evaluation index	Results	Adverse events	Study design
Carlsson et al. [39]	Autologous BM-MSC	Intravenous drip	2.75 × 10^6 cells/kg	12 months	MMTT, AUC, HbA1c, C-peptide, insulin requirements	In response to the MMTT, patients in the control arm had mean decreases in both C-peptide peak values and C-peptide, when calculated as AUC during the 1st year. In contrast, these responses were preserved in MSC-treated patients.	No side effects	Open, single-center, randomized pilot study

Hu et al. [40]	WJ-MSC	Intravenous delivery	1.5–3.2 × 10^7 cells/kg	24 months	FBG, PBG, HbA1c, CPGR, C-peptide, GADA, insulin requirements	Both the HbA1c and C-peptide were significantly better than either pretherapy values or control group patients during the follow-up period.	No obviously adverse reactions	Randomized, double-blind study

Cai et al. [41]	UC-MSC and autologous BM-MNC	Infused through pancreatic artery	1.1 × 10^6 cells/kg	12 months	AUCC-pep, HbA1c, FBG, C-peptide, insulin requirements	The treatment was well tolerated. After 1 year, metabolic measures improved in treated patients.	Transient abdominal pain; bleeding at the puncture site; upper respiratory tract infections	Randomized, controlled, open-label study

MMTT : mixed-meal tolerance test, AUC: FBG: fasting blood glucose, PPG: postprandial blood glucose, CPGR GADA: glutamic acid decarboxylase antibodies, HbA1c: glycosylated hemoglobin, and BM-MNC: bone marrow mononuclear cell.

**Table 3:** Clinical studies of MSC therapy for T2DM.

Publication	Cell resource	Injection method	Injection dose	Number of injections	Follow-up time	Efficacy Evaluation Index	Results	Adverse events	Study design
Liu et al. [42]	WJ-MSC	Splenic artery injection; intravenous injection	1 × 10^6 cells/kg	Twice	12 months	HbA1c, C-peptide, FBG, PBG, insulin requirements, inflammatory markers, T lymphocyte counts	WJ-MSC transplantation significantly decreased the levels of glucose and glycated hemoglobin, improved C-peptide levels and beta cell function and reduced markers of systemic inflammation and T lymphocyte counts.	Fever, subcutaneous hematoma, nausea, vomiting, and headache	Open, single-center, nonrandomized study

Bhansaliet al. [43]	Autologous BM-MSC	Superior pancreatic injection; antecubital vein injection	1 × 10^6 cells/kg	Twice	12 months	Insulin requirements, HbA1c, C-peptide	BM-MSC therapy resulted in a significant decrease in the insulin dose requirement along with an improvement in the stimulated C-peptide levels in T2DM.	No obviously adverse reactions	Randomized, single-blinded, placebo-controlled study

Bhansali et al. [44]	Autologous BM-MSC and autologous BM-MNC	Superior pancreatic injection; antecubital vein injection	MSCs:1 × 10^6 cells/kg MNCs: 1 × 10^9 cells/kg	Twice	12 months	ISI, insulin, HbA1c, C-peptide, HOMA-IR, HOMA-*β*, HOMA-S, GLUT-4, IRS-1	Both autologous BM-MSCs and autologous BM-MNCs resulted in sustained reduction in insulin doses in T2DM and improved insulin sensitivity with MSCs and increased C-peptide response with MNCs.	Nausea and vomiting, local extravasation, minor hypoglycemia	Randomized, single-blinded, placebo-controlled study

Kong et al. [45]	UC-MSC	Vein injection	1 × 10^6 cells/kg	Three times	6 months	FPG, PBG, HbA1c, C-peptide, subsets of T cells	FBG and PBG of the patients in the efficacy group were significantly reduced after UMSC transfusion.	Slight transient fever	Randomized, single-blinded, placebo-controlled study

Chen et al.[46]	UC-MSC	Superior pancreatic injection; antecubital vein injection	1 × 10^6 cells/kg	Four times	6 months	FPG, PBG, HbA1c, C-peptide, HOMA-IR	The FPG, 2hPG, and HbA1c levels were significantly improved in the group with MSCs. Liraglutide treatment in combination with hUC-MSCs improves glucose metabolism and the *β*-cell function in T2DM.	Hypoglycemia event	Randomized, single-blinded, placebo-controlled study

Skyler er al. [47]	Allogeneic BM-MPS	Intravenous infusion	0.3/1/2 × 10^6 cells/kg	Once	12 weeks	HbA1c, FPG	At week 12, the HbA1c target of <7% was achieved, respectively 13.3%, 6.7%, 33.3%; at week 12, the FPG showed no trends across treatment groups.	No serious adverse events	Multicenter, randomized, single-blind, placebo-controlled

Jiang et al. [48]	Placenta-MSCs	Intravenous infusions	1.35 × 10^6 cells/kg	Three times	6 months	Insulin requirements, C-peptide, HbA1c	The daily mean dose of insulin requirements decreased, and the C-peptide level was increased after therapy.		Nonrandomized study

BM-MPCs: bone marrow-derived mesenchymal precursor cells, TNF-*α*: tumor necrosis factor-*α*, ISI: insulin sensitivity index, HOMA-IR: homeostatic model assessment of insulin resistance, HOMA-*β*: homeostatic model assessment of *β*-cell function, HOMA-S: homeostatic model assessment of insulin sensitivity, GLUT-4: glucose transporter type 4, and IRS-1: insulin receptor substrate-1.

## Data Availability

The data in Tables 2 and 3 were derived from http://www.clinicaltrials.gov.
